# Late Presentation of Paediatric Pink Pulseless Supracondylar Fracture of Humerus: A Case Report

**DOI:** 10.5704/MOJ.1911.014

**Published:** 2019-11

**Authors:** RY Kow, JC Yuen, CL Low, KN Mohd-Daud

**Affiliations:** Department of Orthopaedic, Hospital Tengku Ampuan Afzan, Kuantan, Malaysia; * Department of Radiology, Hospital Tengku Ampuan Afzan, Kuantan, Malaysia

**Keywords:** supracondylar, humerus, fracture, pulseless, pink

## Abstract

Supracondylar humeral fracture is the most common elbow injury in children. It may be associated with a vascular injury in nearly 20% of the cases with a pink pulseless limb. We present a unique case of a paediatric pink pulseless supracondylar humeral fracture, seen late, on the 16th-day post-trauma. Open reduction, cross Kirschner wiring, and brachial artery exploration and repair were performed, and the patient recovered well. Early open reduction and exploration of the brachial artery with or without prior CT angiography was a safe approach in treating patients who presented at 16 days.

## Introduction

The supracondylar fracture of the humerus is common among the paediatric population^1, 2^. It accounted for 17.9% of all fractures in children^2^ and commonly presented with the distal metaphyses in extension as a result of a fall on the outstretched hands^2^. Due to the proximity between the proximal fracture fragment and the surrounding soft tissues in extension-type fracture, various neurovascular injuries were often reported^1^. The incidence of nerve injuries had been estimated to be between 12 to 20%, while up to 20% of patients had vascular compromise^2, 3^. In our country, it was not uncommon for patients to present late to the hospital after an injury^4^. Devnani reported a case series of 28 children who sustained supracondylar humeral fractures and sought treatment after a mean of 5.6 days, in the hospital^4^.

We present a case of a late presentation of paediatric pink pulseless supracondylar fracture of the humerus. This was the first case of a delayed presentation of a paediatric pink pulseless supracondylar humeral fracture.

## Case Report

**Fig. 1:**
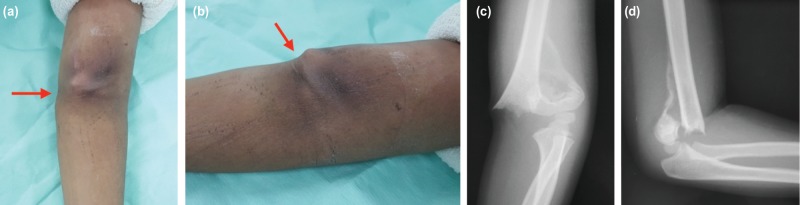
(a, b) shows the metaphyseal spike (red arrow) obtruding the skin at the medial aspect of the antecubital fossa. Brachial and radial pulses are not palpable clinically, and there is no signal detected on hand-held Doppler examination. (c, d) Plain radiographs of the affected left elbow reveal a Gartland III supracondylar humeral fracture with callus formation at the posterior aspect of the proximal fracture fragment.

NB, a six-year old right-hand dominant boy, presented to the hospital complaining of a left elbow swelling and pain for 16 days, pulse has not return after a fall on his left outstretched hand. He denied having any numbness in his left forearm and hand. He came late to the hospital as his parents had first tried alternative medicine treatment after the trauma. On examination, his left elbow was mildly swollen. No wound was noted. There was a hard bony protrusion (red arrow) at the medial aspect of his left elbow (Fig. 1 a,b). The left radial and brachial pulses were absent on palpation and showed no signal with the hand-held Doppler examination, but the capillary refill time was still less than 2 seconds, and the peripheral capillary oxygen saturation (SpO2) was 100% on pulse oximetry. The range of movement of the left elbow was grossly limited due to pain and deformity. Plain radiographs of the left elbow revealed a Gartland III supracondylar humeral fracture with the medial edge of the proximal fragment protruding into the skin (Fig. 1 c,d). There was minimal callus formation at the posterior aspect of the proximal fragment.

**Fig. 2:**
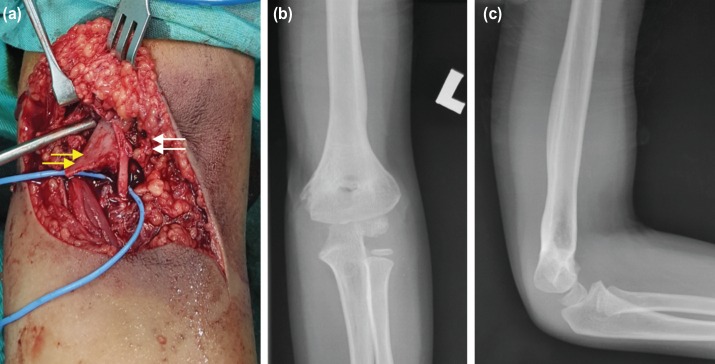
(a) Intra-operatively, the brachial artery is partially lacerated (white arrow) by the sharp edge of the metaphyseal spike (yellow allow). The median nerve appears intact. (b, c) Radiographs of the left elbow (frontal and lateral projections) taken during a final assessment at one year post-operatively showed a well-united fracture.

An early open reduction, with exploration and cross Kirschner wiring, was done without any prior attempt at closed reduction and manipulation. Intra-operatively, the brachial artery was found to be partially transected by the sharp edge of the proximal fracture fragment (Fig. 2 a). There was callus formation at the fracture site with surrounding haematoma. There was no active bleeding from the transected brachial artery. The median nerve was intact. Thrombolysis was performed both in the proximal and distal part of the brachial artery with flushing of heparin saline via a 24 gauge branula. The brachial artery was then repaired with nylon non-absorbable monofilament suture size 7/0. Pulsation of the brachial artery returned after thrombolysis and repair of the brachial artery. After removal of the callus with a rongeur, the humeral supracondylar fracture was carefully reduced and fixed with two crossing Kirschner wires size 1.6mm. Post-operatively, the left elbow was protected with an above-elbow backslab. The left brachial and radial pulses were palpable with good volume, and there was no associated neurological deficit. He was discharged home on post-operative day 3. Daily pin site dressing was carried out at the health clinic. The protective backslab and Kirschner wires were removed at three weeks postoperatively. He was then referred for physiotherapy and rehabilitative exercises. He was followed-up at the orthopaedic clinic for a year. During the final assessment, the fracture site had united and remodelled well (Fig. 2 b,c) with excellent cosmetic and functional outcomes based on Flynn’s criteria. No complication such as Volkman's contracture was noted.

## Discussion

In displaced supracondylar humeral fractures, the brachial artery is the most vulnerable, often stretched or kinked by the displaced fracture fragments^3^. The brachial artery is at greater risk due to the ulnar-sided tether of the supratrochlear artery^3^. There is also a risk of direct injury to the brachial artery, with a contusion, compression by the adjacent soft tissues, or an intimal injury, with partial laceration or even a complete transection^3^. Impaired blood supply to the distal part of the upper extremity could lead to daunting complications such as Volkmann’s ischemia if it were not recognised and treated promptly^1^. In the patient, the sharp edge of the proximal fracture fragment caused a partial laceration of the brachial artery, leading to a pulseless limb. There was formation of multiple collateral blood vessels, bypassing the major artery to supply the distal hand, and thus preventing ischemic gangrene of the upper limb.

Apart from the clinical assessment, various tools and imaging methods such as Doppler ultrasound and angiography could aid in a detailed vascular assessment. Doppler ultrasound could be performed rapidly at the bedside for vascular assessment and estimation of the severity of the vascular injury. Computed tomography (CT) angiography was not necessary in this case as it did not provide additional benefit as the site of vascular injury could be easily identified and located at the fracture site intra-operatively^3^. CT angiography still played a role in cases where pre-operative planning was needed, such as in complicated injuries with comminuted fractures or suspected segmental artery injuries.

In a patient with vascular compromise due to a displaced supracondylar humeral fracture, the consensus was to track and reduce the fracture^3^ gently. The manoeuvre of flexing the elbow up to 45 degree and gentle traction could relieve the pressure from the anterior structures, potentially separating the sharp edges of the proximal fracture fragment from the neurovascular structures, hence improving the perfusion^1, 3^. If the fracture was not reduced, and there was a vascular compromise, an open reduction and exploration of the brachial artery would be indicated (Fig. 3). Similarly, if the vascular assessment showed a pale and pulseless upper limb, an open reduction and exploration of the artery would also be indicated. In the patient, it was not advisable to perform any manipulation as it might damage the remaining collateral blood vessels which were supplying the upper limb distally. Furthermore, the presence of callus noted on plain radiographs showed that it was almost impossible to reduce the fracture properly. Hence, we believed open reduction and exploration as the best approach in this situation.

Multiple surgical approaches had been described to treat paediatric supracondylar humeral fractures^5^. An anterior approach was used in this case due to the presence of a protruding metaphyseal spike from the proximal fracture fragment at the medial aspect. Moreover, the brachial artery and median nerve were easily accessible via this approach. A “lazy S” incision was made at the flexion crease of the antecubital fossa, crossing the bony spike area to release the soft tissue that was hinging on the bony spike. The incision was also extended medially to identify the brachial artery and the median nerve. The callus at the posterior aspect was carefully removed with a rongeur to facilitate the reduction of the fracture.

In conclusion, the delayed presentation of a paediatric pink pulseless supracondylar fracture of the humerus was extremely rare. Early open reduction and exploration of the brachial artery with or without prior CT angiography was a safe approach in treating patients who presented late.
